# Investigation of a new lead-free Bi_0.5_(Na_0.40_K_0.10_)TiO_3_-(Ba_0.7_Sr_0.3_)TiO_3 _piezoelectric ceramic

**DOI:** 10.1186/1556-276X-7-24

**Published:** 2012-01-05

**Authors:** Pharatree Jaita, Anucha Watcharapasorn, Sukanda Jiansirisomboon

**Affiliations:** 1 Department of Physics and Materials Science, Faculty of Science, Chiang Mai University, Chiang Mai, 50200, Thailand; 2 Materials Science Research Center, Faculty of Science, Chiang Mai University, Chiang Mai, 50200, Thailand

**Keywords:** ceramics, X-ray diffraction, dielectric properties, microstructure, piezoelectricity

## Abstract

Lead-free piezoelectric compositions of the (1-*x*)Bi_0.5_(Na_0.40_K_0.10_)TiO_3_-*x*(Ba_0.7_Sr_0.3_)TiO_3 _system (when *x *= 0, 0.05, 0.10, 0.15, and 0.20) were fabricated using a solid-state mixed oxide method and sintered between 1,050°C and 1,175°C for 2 h. The effect of (Ba_0.7_Sr_0.3_)TiO_3 _[BST] content on phase, microstructure, and electrical properties was investigated. The optimum sintering temperature was 1,125°C at which all compositions had densities of at least 98% of their theoretical values. X-ray diffraction patterns that showed tetragonality were increased with the increasing BST. Scanning electron micrographs showed a slight reduction of grain size when BST was added. The addition of BST was also found to improve the dielectric and piezoelectric properties of the BNKT ceramic. A large room-temperature dielectric constant, *ε*_*r *_(1,609), and piezoelectric coefficient, *d*_33 _(214 pC/N), were obtained at an optimal composition of *x *= 0.10.

## Background

Although Pb(Zr, Ti)O_3 _has played a dominant role in piezoelectric materials, waste of products containing Pb causes a crucial environmental problem. Thus, it is urgent to search for lead-free piezoelectric ceramics with excellent properties comparable to those found in lead-based ceramics.

Because it has a large remanent polarization [*P*_*r*_] of approximately 38 μC/cm^2 ^and a high Curie temperature [*T*_*c*_] of approximately 320°C, (Bi_0.5_Na_0.5_)TiO_3 _[BNT] is a candidate material for a lead-free piezoelectric ceramic. However, poling difficulties due to its high coercive field [*E*_*c*_] of approximately 73 kV/cm and high conductivity often require some modifications. It has been reported that BNT-based compositions modified with BaTiO_3 _[1], (Ba, Sr)TiO_3 _[2], and Ba(Zr, Ti)O_3 _[3] showed improved piezoelectric properties. Another modification based on the work of Sasaki et al. [4] showed that Bi_0.5_(Na_1-*x*_K_*x*_)_0.5_TiO_3 _ceramic had a morphotropic phase boundary [MPB] between rhombohedral and tetragonal phases near *x *= 0.16 to 0.20, at which a relatively high *d*_33 _of 151 pC/N was obtained.

Aside from BNT, lead-free barium strontium titanate, (Ba_1-*x*_Sr_*x*_)TiO_3_, as well as doped BaTiO_3 _are currently important dielectric materials for capacitor applications [5]. The main purpose of adding Sr^2+ ^into BaTiO_3 _is to shift the *T*_*c *_(approximately 130°C) towards room temperature, offering a high dielectric constant and a low dielectric loss, tan*δ *[6]. At *x *= 0.3 composition, a relatively high permittivity was achieved. Recently, Lee et al. [2] have studied the (1-*x*)(Bi_0.5_Na_0.5_)TiO_3_-*x*(Ba_0.7_Sr_0.3_)TiO_3 _system. The addition of (Ba_0.7_Sr_0.3_)TiO_3 _into (Bi_0.5_Na_0.5_)TiO_3 _generated a phase transition from rhombohedral to tetragonal. The improvement of both dielectric and piezoelectric performances was found at an MPB of *x *= 0.08.

In order to develop a new material system with both high piezoelectric and dielectric performances, (1-*x*)Bi_0.5_(Na_0.40_K_0.10_)TiO_3_-*x*(Ba_0.7_Sr_0.3_)TiO_3 _[(1-*x*)BNKT-*x*BST] (*x *= 0 to 0.20) ceramics were prepared. The effect of the BST concentration on phase, microstructure, and electrical properties of the ceramics was investigated and discussed.

## Methods

Conventional mixed-oxide technique was used to prepare Bi_0.5_(Na_0.40_K_0.10_)TiO_3 _and (Ba_0.7_Sr_0.3_)TiO_3 _powders. The starting materials were Bi_2_O_3_, Na_2_CO_3_, TiO_2_, K_2_CO_3_, BaCO_3_, and SrCO_3_. A stoichiometric amount of BNKT and BST powders was weighed, ball-milled for 24 h, and dried using the oven-drying method. BNKT and BST powders were separately calcined for 2 h at 900°C for BNKT and 1,100°C for BST. The calcined powders were then weighed, mixed, and oven-dried to produce the mixed powders of (1-*x*)BNKT-*x*BST (when *x *= 0, 0.05, 0.10, 0.15, and 0.20). After drying and sieving, a few drops of 3 wt.% PVA binders were added before being uniaxially pressed into pellets of 10 mm in diameter. These pellets were covered with their own powders and subsequently sintered at 1,050°C to 1,175°C for 2 h with a heating/cooling rate of 5°C/min.

Phase evolution was examined using an X-ray diffraction [XRD] diffractometer (X'Pert, PANalytical B.V., Almelo, The Netherlands). Bulk densities were determined using Archimedes' method. The theoretical densities of all samples were calculated based on the theoretical densities of BNKT (5.84 g/cm^3^) [7] and BST (5.75 g/cm^3^) [8]. Surfaces of the ceramics were observed using a scanning electron microscope [SEM] (JSM-6335F, JEOL Ltd., Akishima, Tokyo, Japan). Grain size was determined by mean linear intercept method.

For electrical measurements, two parallel surfaces were polished and painted with silver paste for electrical contacts. Dielectric properties were determined at 25°C to 500°C with a frequency of 10 kHz using a 4284A-LCR meter (Agilent Technologies, Santa Clara, CA, USA) connected to a high-temperature furnace. A standard Sawyer-Tower circuit was used to measure the hysteresis loop. The samples were poled at 60°C in a stirred silicone oil bath by applying a DC electric field of 5 kV/mm for 15 min, and piezoelectric measurements were then carried out using a *d*_33_-meter (S5865, KCF Technologies, Inc., State College, PA, USA).

## Results and discussion

**Figure 1:**
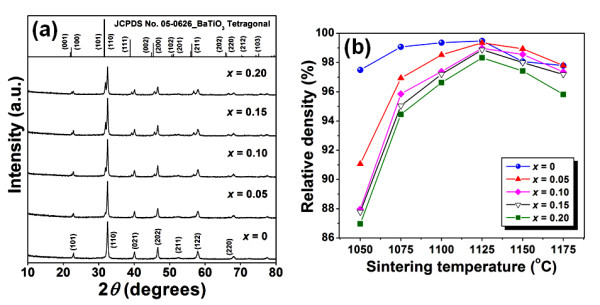
**XRD patterns and plots**. (**a**) XRD patterns of BNKT-BST powders. (**b**) Plots of relative density and sintering temperature.

XRD patterns of BNKT-BST mixed powders are shown in Figure 1a. There were no detectable impurities for all compositions. A separation of (110) main peak (2*θ *at approximately 32°) was not observed for pure BNKT powder. When 5 mol% BST was added, the (110) main peak was slightly asymmetrical and featured a slight splitting of the BST peak. With increasing BST, peaks that belonged to BST were dominantly shown and led to more splitting.

Plots of relative density as a function of sintering temperature with different BST contents are shown in Figure 1b. The optimum sintering temperature of BNKT-BST ceramics was 1,125°C at which all samples had densities ranging from 5.72 to 5.81 g/cm^3^, corresponding to at least 98% of their theoretical values (see Table 1). Thus, the samples sintered at this temperature were selected for further characterizations.

**Table 1:** Physical and electrical properties of (1-*x*)BNKT-*x*BST ceramics sintered at 1,125°C

*x*	Density (g/cm^3^)	*c/a*	Grain size (μm)	*T*_*c *_(°C)	*ε*_*r*_^a^	tan*δ*^a^	*P*_*r *_(μC/cm^2^)	*E*_*c *_(kV/cm)	*R*_*sq*_	*d*_33_ (pC/N)
0	5.81 ± 0.01	1.0083	0.60 ± 0.09	320	1,419	0.0479	31.62	32.01	1.12	178
0.05	5.80 ± 0.02	1.0118	0.40 ± 0.04	310	1,581	0.0559	14.03	10.28	0.48	98
0.10	5.77 ± 0.01	1.0120	0.39 ± 0.04	308	1,609	0.0618	28.14	22.96	1.04	214
0.15	5.76 ± 0.01	1.0156	0.46 ± 0.07	305	1,430	0.0576	25.11	25.67	0.98	205
0.20	5.72 ± 0.01	1.0163	0.47 ± 0.06	289	1,082	0.0477	21.96	29.08	0.98	191

^a^Dielectric data obtained at room temperature (1 kHz).

**Figure 2:**
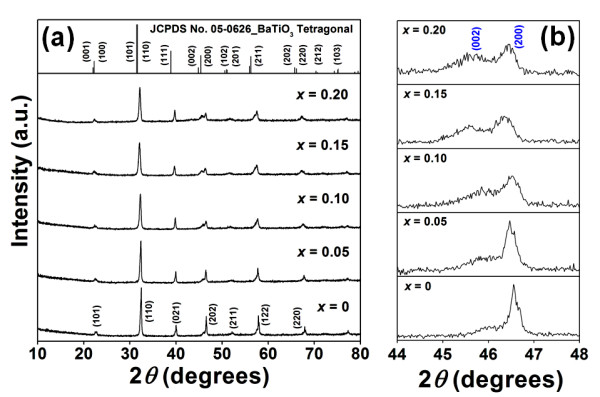
**XRD patterns of BNKT-BST ceramics**. The samples were sintered at 1,125°C. (**a**) 2*θ *= 10° to 80° and (**b**) 2*θ *= 44° to 48°.

Figure 2a showed XRD patterns of BNKT-BST ceramics. All compositions showed a pure perovskite phase. BST had diffused into the BNKT lattice and formed solid solutions. All peaks were found to shift slightly to a lower angle. The shift scale was increased with the increasing of BST to a maximum value at *x *= 0.20. The slight distortion of the XRD patterns was attributed to larger sized Ba^2+ ^(1.42 Å) and Sr^2+ ^(1.26 Å) ions diffused into the BNKT lattice to replace Bi^3+ ^(1.17 Å), Na^+ ^(1.18 Å), and K^+ ^(1.33 Å) [9], resulting in the enlargement of lattice constant and lattice energy which induced a phase transformation in order to stabilize the structure [10]. In Figure 2b, Bi_0.5_(Na_0.40_K_0.10_)TiO_3 _was a mixed phase between BNT rhombohedral and BKT tetragonal, whereas (Ba_0.7_Sr_0.3_)TiO_3 _was mainly tetragonal in phase. At *x *= 0.05, the peak around 46.5° was slightly asymmetrical, and (202) peak started to split into two peaks of (002) and (200). At *x *= 0.10, the intensity of (200) peak was found to decrease, while it was gradually increased for (002) peak. Moreover, (200) peak underwent an asymmetric broadening, and (002) peak obviously split into two peaks. This indicated that the addition of a higher tetragonal BST into BNKT at *x *= 0.10 became close to the optimum of rhombohedral and tetragonal phases of the BNKT-BST system. As its crystal structure was considered to contain nearly the same amount of coexisting rhombohedral and tetragonal structures in BNKT-0.10BST ceramic, the optimal dielectric and piezoelectric properties should be obtained in this composition. The addition of a BST content greater than 10 mol% led to a wider separation of the (002) and (200) peaks and showed mainly a tetragonal structure, corresponding to an increase in tetragonality as shown in Table 1.

**Figure 3:**
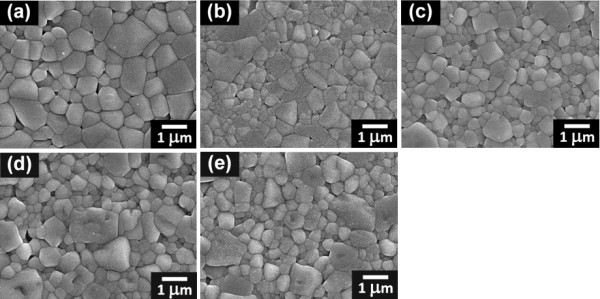
**SEM micrographs of (1-*x*)BNKT-*x*BST ceramics**. The samples were sintered at 1125°C. (**a**) *x *= 0, (**b**) *x *= 0.05, (**c**) *x *= 0.10, (**d**) *x *= 0.15, and (**e**) *x *= 0.20.

SEM images in Figure 3 confirmed that all ceramics were of high quality and densely sintered at 1,125°C. An addition of BST allowed shortening of the sintering duration to attain a dense sintered bulk with similar grain size. The microstructure of a pure BNKT ceramic revealed a larger grain size (0.60 μm) with a relatively wide grain size distribution compared to BST-added samples. The addition of BST, however, slightly inhibited grain growth, as can be seen from a slight drop of grain size from 0.60 μm for pure BNKT to around 0.39 to 0.47 μm for BST-added samples (see Table 1).

**Figure 4:**
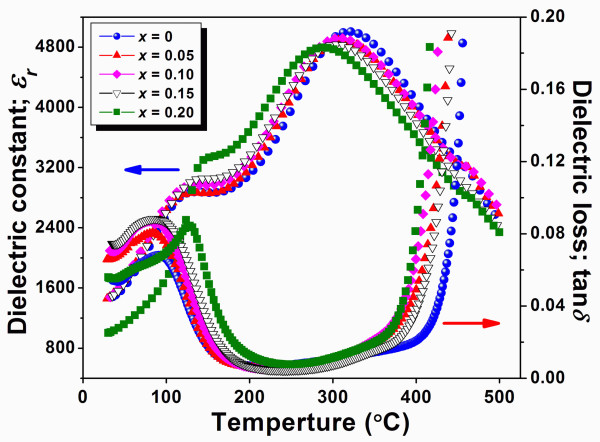
**Plots of temperature dependence on dielectric constant and dielectric loss**. The measurement was done at a frequency of 10 kHz for BNKT-BST ceramics and sintered at 1,125°C.

Dielectric constant and dielectric loss of (1-*x*)BNKT-*x*BST ceramics were plotted as a function of temperature shown in Figure 4. At *T*_*c*_, the highest *ε*_*r *_of 5,006 was observed in pure BNKT. For BST-added samples, the maximum *ε*_*r *_of 4,921 was observed in BNKT-0.10BST ceramic. Since the crystalline structure of BNKT-0.10BST was considered to be near optimum composition having a comparable coexistence of rhombohedral and tetragonal phases, the increase in *ε*_*r *_would be expected. The *T*_*c *_of pure BNKT was found to be 320°C. It has been shown that an A-site isovalent additive had the effect of lowering the *T*_*c *_[11]. BST is virtually an A-site isovalent additive in which Ba_0.7_Sr_0.3 _has an effective charge of +2, which is the same as +2 of Bi_0.5_(Na_0.40_K_0.10_). Moreover, BST has a much lower *T*_*c *_(approximately 42°C) [12] compared with BNKT; a reduction of *T*_*c *_was observed in our system. At room temperature, *ε*_*r *_of pure BNKT was found to be 1,419. The addition of 10 mol% BST showed an optimum *ε*_*r *_of 1,609. As free energy of the rhombohedral phase was close to that of the tetragonal phase, these two phases existing at the BNKT-0.10BST composition easily changed to each other when an electric field was applied. This helped promote the movement and polarization of ferroelectric active ions, leading to the increase of *ε*_*r *_[13]. With a further increasing BST, a slight decrease in *ε*_*r *_was observed. Phase analysis using XRD patterns indicated that the compositions slightly deviated from the optimal composition, and hence, the lowering of *ε*_*r *_values in our samples seemed reasonable.

**Figure 5:**
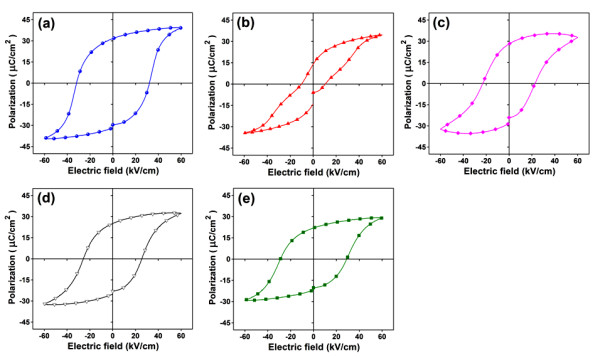
**Plots of polarization as an electric field function of (1-*x*)BNKT-*x*BST ceramics**. The samples were sintered at 1,125°C. (**a**) *x *= 0, (**b**) *x *= 0.05, (**c**) *x *= 0.10, (**d**) *x *= 0.15, and (**e**) *x *= 0.20.

From Figure 5, the hysteresis loop of pure BNKT showed the maximum *E*_*c *_at approximately 31.49 kV/cm, *P*_*r *_at approximately 30.48 μC/cm^2^, and *R*_*sq *_at approximately 1.10. Ferroelectric property was slightly degraded when BST was added, as can be seen from a decreasing trend in *R*_*sq*_, *E*_*c*_, and *P*_*r*_. Since BST by itself was known to have a low *E*_*c *_(approximately 2 kV/cm) and *P*_*r *_(approximately 5 μC/cm^2^) [12] compared with pure BNKT, this seemed to be the reason for a reduction of both *P*_*r *_and *E*_*c *_observed in BST-added samples. Among BST-added samples, the highest *P*_*r *_of 28.14 μC/cm^2 ^was observed for BNKT-0.10BST. Besides, an increase of spontaneous polarization directions due to the coexistence of rhombohedral and tetragonal phases (eight directions for rhombohedral phase and six directions for tetragonal phase) was also a reason that gave a high *P*_*r *_in BNKT-0.10BST. Moreover, a decrease of *E*_*c *_(approximately 22.96 kV/cm) in BNKT-0.10BST in comparison with that in pure BNKT was also observed at this composition. This decrease in *E*_*c *_indicated easier ionic motion, and therefore, the improvement of piezoelectricity would be expected for this composition [14]. The addition of BST content over 10 mol% caused the material to completely transform to a tetragonal phase, resulting in a slight decrease of *P*_*r*_. The reduction of *P*_*r *_when the crystal structure changed to be more tetragonal in structure was similar to the previous work on BNT-BST system [2].

Piezoelectric coefficients of (1-*x*)BNKT-*x*BST ceramics are listed in Table 1. The *d*_33 _of pure BNKT ceramic was 178 pC/N, which was close to the value of 165 pC/N observed earlier by Hiruma et al. [15]. The highest *d*_33 _of 214 pC/N was observed for the BNKT-0.10BST ceramic. As the crystal structure of BNKT-0.10BST was nearly a coexistence of rhombohedral and tetragonal phases, a flexibility increase in the domain wall could effectively occur. Moreover, *E*_*c *_of this composition was lower than that of pure BNKT, whereas *P*_*r *_was maintained. Thus, it is obvious that the optimal piezoelectric properties would occur in this composition. The *d*_33 _decreased with the further increasing BST content of over 10 mol%. This was supported by phase analysis using XRD which indicated a deviation of the composition from the mixed rhombohedral and tetragonal phases of BNKT-BST system to mainly the tetragonal BST phase. In addition, the change in crystal structure to being more tetragonal may also contribute to the reduction in the piezoelectric performance of BNKT-BST ceramics similar to the reduction in *d*_33 _observed in the previous work on BNKT-BZT system [13].

## Conclusions

New (1-*x*)BNKT-*x*BST ceramics were successfully fabricated. The optimum sintering temperature of all ceramics was 1,125°C. XRD indicated that the addition of BST into BNKT caused a change in crystal structure and increase in lattice parameters. The addition of BST also inhibited grain growth. The incorporation of 10 mol% BST was found to be an optimum condition that could enhance *ε*_*r *_and *d*_33 _to the maximum values of 1,609 and 214 pC/N, respectively. In addition, it also possessed a relatively low *E*_*c*_, while *T*_*c *_and *P*_*r *_were quite comparable to that of pure BNKT. Therefore, BNKT-0.10BST ceramic is a promising candidate as a new lead-free piezoelectric ceramic which can be further used in actuator applications.

## Competing interests

The authors declare that they have no competing interests.

## Authors' contributions

PJ carried out experiments and wrote the manuscript. AW and SJ participated in the conception of the study and revised the manuscript for important intellectual contents. All authors read and approved the final version of the manuscript.
